# Surface-Enhanced Raman Scattering Spectroscopy and Microfluidics: Towards Ultrasensitive Label-Free Sensing

**DOI:** 10.3390/bios8030062

**Published:** 2018-06-29

**Authors:** Krishna Kant, Sara Abalde-Cela

**Affiliations:** International Iberian Nanotechnology Laboratory (INL), Avda Mestre José Veiga, 4715-310 Braga, Portugal; krishna.kant@inl.int

**Keywords:** microfluidics, surface-enhanced Raman scattering, label-free

## Abstract

Raman scattering and surface-enhanced Raman scattering (SERS) spectroscopy have demonstrated their potential as ultrasensitive detection techniques in the past decades. Specifically, and as a result of the flourishing of nanotechnology, SERS is nowadays one of the most powerful sensing techniques, not only because of the low detection limits that it can achieve, but also for the structural information that it offers and its capability of multiplexing. Similarly, microfluidics technology is having an increased presence not only in fundamental research, but also in the industry. The latter is because of the intrinsic characteristics of microfluidics, being automation, high-throughput, and miniaturization. However, despite miniaturization being an advantage, it comes together with the need to use ultrasensitive techniques for the interrogation of events happening in extremely small volumes. The combination of SERS with microfluidics can overcome bottlenecks present in both technologies. As a consequence, the integration of Raman and SERS in microfluidics is being investigated for the label-free biosensing of relevant research challenges.

## 1. Introduction

Raman spectroscopy and surface-enhanced Raman scattering (SERS) spectroscopy have been flourishing in recent years as powerful analytical techniques. Despite the discovery of Raman spectroscopy at the beginning of the 20th century and SERS during the 1970s, their application in solving real world problems only started booming in the recent years, closely linked to the advances in nanotechnology [1,2]. Nowadays, Raman and SERS are established among the most powerful analytical tools demonstrating their sensing potential in various fields such as environmental and water pollution, pharmacology, food, biology, medicine, forensics, arts, homeland security, and chemical weapons [3,4,5,6,7,8,9]. Raman and SERS are vibrational analytical techniques, reporting on the inelastic scattering phenomena of molecular bonds when irradiated with light. Thus, Raman-based analytical techniques provide the molecular fingerprint of the molecule under study as an outcome. SERS spectroscopy is currently more extended than Raman scattering when facing more complex analytical problems, because of the characteristic of a low cross-section of conventional Raman scattering. This low cross-section of Raman (only 1 photon out of 10^6^–10^8^ undergoes Raman scattering) can be overcome by using nanoparticles to enhance this Raman efficiency [10]. Metallic nanoparticles and nanostructures function as *nanoantennas*, enhancing the Raman signal of the molecules in the vicinity of the metallic nanostructure. This phenomenon has been explained and thoroughly studied in the 1970s and 1980s, being the result of the combination of two different mechanisms: the electromagnetic (EM) and the chemical mechanism or chemical transfer mechanism (CT) [11,12,13]. The EM is the major contributor to the enhancement, and arises from the field enhancement of both incident and scattered fields as a consequence of the localized surface plasmon resonance (LSPR) excitation. The CT is the contribution from the molecular structure of the analyte and specific interactions between the analyte and the nanostructure. Enhancement factors up to 11–12 order of magnitude for average SERS and of 14 orders of magnitude for single molecule SERS, when compared with conventional Raman, have been reported [14,15]. The design of nanostructured materials for their use as SERS substrates has attracted intensive attention at the end of the 20th and the beginning of the 21st century [16,17]. However, the actual trend is to apply the generated knowledge on SERS substrates for the detection of relevant species in different matrices and combine it with novel key enabling technologies [18,19].

Microfluidics technology is based in the handling of liquids in confined environments in the microscale range. Because of the confinement in the microscale, different properties than when compared with classical fluid mechanics arise. Those properties can be exploited to develop novel and powerful technologies. Microfluidics offers automation, miniaturization, reduced sample volumes, and improved energy and heat conversion, among others [20]. The miniaturization of large laboratory equipment and processes offers substantial benefits over traditional methodologies, being more cost effective and offering automation. Altogether, the application of microfluidics technology was expanded to several fields of research and industry. Microfluidics technology demonstrated to be useful for material synthesis and discovery, process automation, lab-on-a-chip (LoC) and point-of-care (PoC) sensing, and biosensing devices in different research areas [20,21,22,23]. Microfluidic systems integrated with different functional units (e.g., pumps, valves, actuators, reactors, etc.) can work as a function unit for analytical detection of complex samples [23]. Transport of fluid in these miniaturized systems can be done either in passive mode as zero powered device by use of gravity and capillary forces, or in an active mode by using microfluidic pumps, centrifugal forces, electric power, and so on [24]. Microfluidic devices are usually low cost and easy to fabricate at high throughput compared with conventional systems. However, despite all the benefits of using microfluidics, because of the small volume handled, highly sensitive analytical techniques need to be integrated. There are several high-throughput detection techniques that have been successfully combined with microfluidic platforms: laser-induced fluorescence (LIF), mass spectrometry (MS), nuclear magnetic resonance (NMR), isothermal amplification for DNA detection, and so on [25]. All of these techniques provided promising results regarding sensitive detection in a microfluidic channel or reservoir, LIF being the most used and representative technique, as a result of its ultra-sensitivity, low cost, and ease of use. Nevertheless, LIF presents some limitations, including the non-native fluorescence of all analytes (making labelling compulsory), the width of the fluorescence peaks that can lead to overlaps when a multiplex detection is being performed, or photobleaching effects.

Nowadays, SERS provides a sensitivity that is comparable with fluorescence detection and is also known as a promising tool for application in multiplex technologies [18]. SERS detection is usually carried out statically, with analytes placed on the surface of solid thin films or on colloidal particles in solution, one by one. The combination of SERS and microfluidic sensors is meaningful for both SERS and microfluidics technology [26,27,28]. SERS acquisition under flowing conditions prevents variable mixing times, variable scattering geometries, localized heating, and photo-dissociation. Thus, for on-chip SERS detection in a fluidic channel, it is known that flow conditions yield more reproducible results than static conditions because of the more consistent geometries and heat dissipation properties [28]. Furthermore, the combination of the great intensity of the signals obtained with this technique with the latest developments in the fields of microfluidics and optical spectroscopy (2D charge-coupled devices (CCDs)), enables real time (i.e., online) monitoring of continuous flows. In fact, online SERS detection has been already demonstrated using microfluidic devices for a number of analytes including pesticides and metals in water, explosives and pollutants, drugs, cancer cells and metabolites, and many others [26,29,30,31,32,33]. Therefore, the concurrent rise of SERS and microfluidic technologies in recent years is more of an expected result than coincidence. 

## 2. Considerations

**Figure 1:**
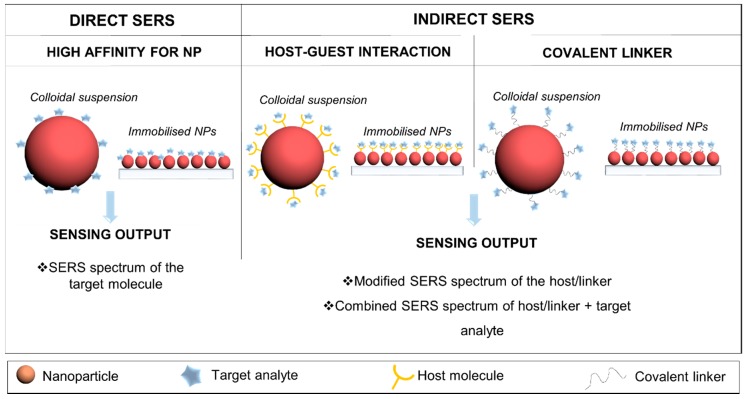
Label-free strategies for surface-enhanced Raman scattering (SERS) detection classified as direct and indirect. The direct detection is used when the target analyte has high affinity to the nanoparticle. Indirect strategies using host molecules or covalent linkers are used to capture analytes with low affinity for the nanoparticle with high specificity.

For practical quantitative analytical applications, SERS must fulfil the typical requirements of an analytical technique: reproducibility of results, linearity of the response, standardization, molecular selectivity, and clear methodology for sample preparation. However, the task of finding “the universal SERS substrate” is not trivial and may not even be the right approach for implementing SERS as an analytical tool. As a consequence, and in order to maximize the EF of SERS compared with conventional Raman, rational design of SERS substrates first considering the analytical challenge should be taken into account. Upon thorough analysis of the analytical problem, SERS substrate design, detection strategy and architecture for sensing need to be considered for different applications [17]. SERS substrates have been mainly obtained in electrodes, nanostructured films, and colloidal nanoparticles. Plasmonic colloidal nanoparticles (Au, Ag, Cu, Pt, TiO_2_, Al, etc.) have been the most used SERS substrates by far [17,18]. As SERS is mainly dominated by the electromagnetic properties of the plasmonic nanostructures, being size, shape and composition, they are the most important triggering factors for the enhancement of the Raman signal [17]. In terms of composition, Ag and Au are among the most preferred choice by researchers, and they have been used in different fashions depending on the application. The most straightforward path is using metallic nanoparticles in colloidal dispersion, controlling their size and shape [10]. Plasmonic Au and/or Ag nanoparticles (NPs) between 30 and 100 nm arise as the most powerful SERS enhancers. Further, when those NPs have non-spherical shapes or sharp tips (nanostars, nanourchins, nanorods, etc.), the SERS effect can be further boosted [34]. Nevertheless, dispersed colloidal nanoparticles have low stability and tend to aggregate, and also many molecules have no affinity for the Au or Ag surface [35]. Further, an extra enhancement of the Raman signal has been described at the so-called *hot spots*, which are formed by the coupling of the electromagnetic fields of NPs separated by a short distance (2–5 nm) [36]. In order to overcome the disadvantages of using dispersed NPs and to take advantage of the *hot spots* extra enhancement, several strategies have been proposed in the most recent years, such as the controlled self-assembly of plasmonic NPs [37], the use of standard nanolithography (nanopillars, nanoneedles, bifunctional NPs) fabrication [34], or the use of hybrid materials combining polymers or hydrogels as support the plasmonic NPs [38]. Specific reviews have been published elsewhere expanding on the types, fabrication strategies, and applications of SERS substrates [17,18,39]. As mentioned above, one of the most important advantages is that Raman and SERS are inherently label-free detection methods, even though labelling strategies can also be applied when required. When using SERS, the detection of the molecule under study can be done in different fashions (Figure 1) that can be classified as direct (always falling into label-free category) or indirect (label or non-label free) strategies [39]. When following a direct approach, the target molecule is in close contact with the nanosurface and the acquired spectrum is that of the target molecule. This method is used when the molecule under study has affinity for the surface of the nanostructure (usually gold or silver). Additionally, if the molecule has no affinity for the nanosurface, optical accumulators arise as an alternative approach. Optical accumulators benefit from the physical properties of the material (magnetic accumulators, hydrogels, etc.) to bring the analyte close to the NP [40,41,42]. On the other hand, indirect strategies can be classified as (i) host-guest or linker-mediated strategies (label-free), where the target analyte induces a change in host molecules close to the nanoparticle and the change in the spectrum of the host or linker relates to the presence of the analyte [38,43,44,45,46,47]; (ii) nanostructures can be codified with known Raman probes and further functionalized to capture the target analyte [48,49,50]. The latter strategy is the one considered as non-label free in the SERS context, and it will not be covered in this review as it has been already reviewed elsewhere [51,52].

When coupling SERS sensing with microfluidic platforms, there are additional considerations such as instrument integration, static or in-flow measurements, immobilized nanoparticles, or nanoparticles in colloidal dispersion, among others. Although, independently on the followed strategy, the most important factor when designing a label free SERS-based optofluidic platform is to achieve effective mixing/contact between the SERS substrate and the analyte under study. Current mixing techniques can be classified as active mixing and passive mixing [53]. Microfluidics active mixing techniques, such as electro-kinetic, acoustic, dielectrophoretic, pressure, thermal, or magnetically driven mixers, require external energy supply and usually costly external components to be added to the microfluidic devices [54]. In contrast, as passive mixing only needs a well-designed microfluidic channel (e.g., zigzag-shaped channel, 3D serpentine, slanted wells, surface-chemistry technology, etc.), it is more extensively used compared with active mixing for SERS microfluidic systems.

**Figure 2:**
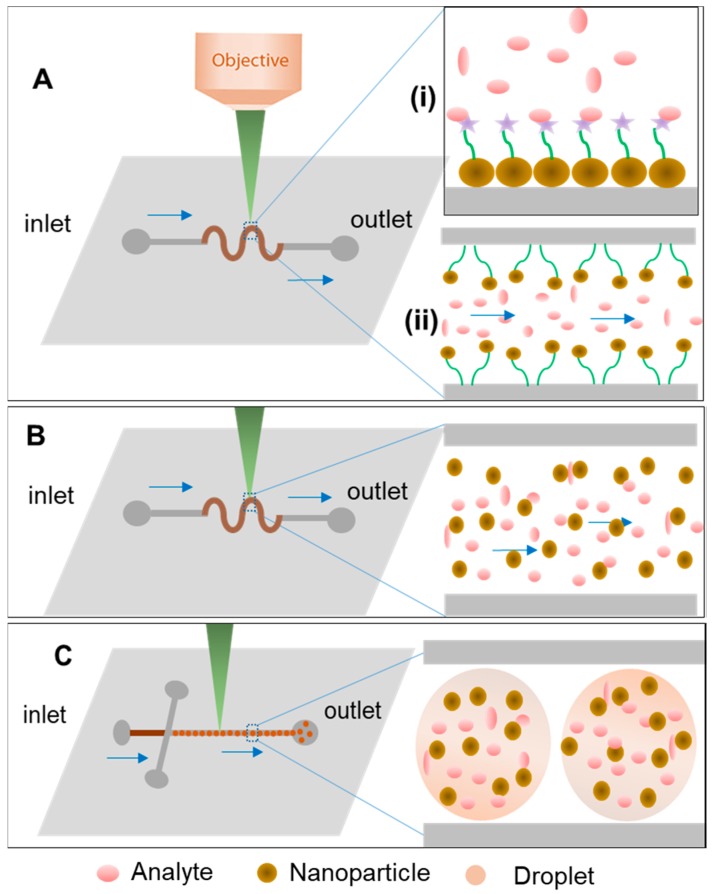
Schematic illustration of different strategies for lab-on-a-chip (LOC)-SERS devices; (**A**) immobilization of NPs in microfluidic channels in (i) static or (ii) continuous flow approach; (**B**) colloidal dispersions and continuous flow approach; and (**C**) in segmented flow.

In SERS microfluidic systems, the analyte fluids are controlled and analyzed within the microchannel, in which they interact with externally added nanoparticles or built in micro or nanostructures of noble metal [55]. Microfluidic strategies can be generally divided in two types of platforms, based on continuous flow or segmented flow, the latter of which is also known as droplet-based microfluidics [56]. The use of SERS has been demonstrated in both types of microfluidic devices. On one hand, continuous SERS detection allows for highly-reproducible measurement with the main advantage of avoiding sample heating and scattering geometries dependence as the nanoparticles being the SERS substrates are in suspension (Figure 2B) [57,58]. However, molecular diffusion, which controls mixing in microfluidic channels, also plays a role in SERS detection because of the molecular displacement at the detection site [59]. Whenever the latter approach is followed, channel design and geometry promoting an efficient mixing of nanoparticles and analytes are extremely important, affecting the reproducibility and sensitivity of SERS detection [60]. Still based in continuous flow platforms, instead of injecting pre-formed NPs to flow along the channels with the sample, NPs can be immobilized and built into the microfluidic channels or in sensing areas (chambers or reservoirs) and even synthesized in situ in the microfluidic device, as shown in Figure 2A [61]. Microfluidic SERS devices with built-in SERS substrates have several advantages of low-dosage and high-sensitivity [62]. This strategy also allows for the integration of many pre-treatment steps, such as mixing or concentration of sample, for enhanced SERS outputs [62]. Thus, fabrication of SERS-active nanostructures and their integration on microfluidic chips is crucial and can be developed from zero-dimensional noble metal nano-spots to 3D nano-pillars or multidimensional star-shaped nanoparticles [27,37,63]. On the other hand, droplet-based microfluidics (Figure 2C) benefits from the immiscibility of two phases to encapsulate the SERS substrates in microdroplets (acting as microreactors) that can be analysed separately without having the diffusion effect in the flow channel, and at a high-throughput [53]. Herein, the recent advancement in microfluidic SERS devices, especially the combined forms of SERS-active nanostructures and externally injected nanomaterial are a broad area that has been reviewed elsewhere [26,28,64]. In this review, we expanded on the most recent development of label-free SERS detection in microfluidic devices using different strategies from the sensing material and microfluidics approach point of view.

## 3. SERS and Microfluidics for Label-Free Detection

### 3.1. Dispersed Nanoparticles and Continuous Flow

**Figure 3:**
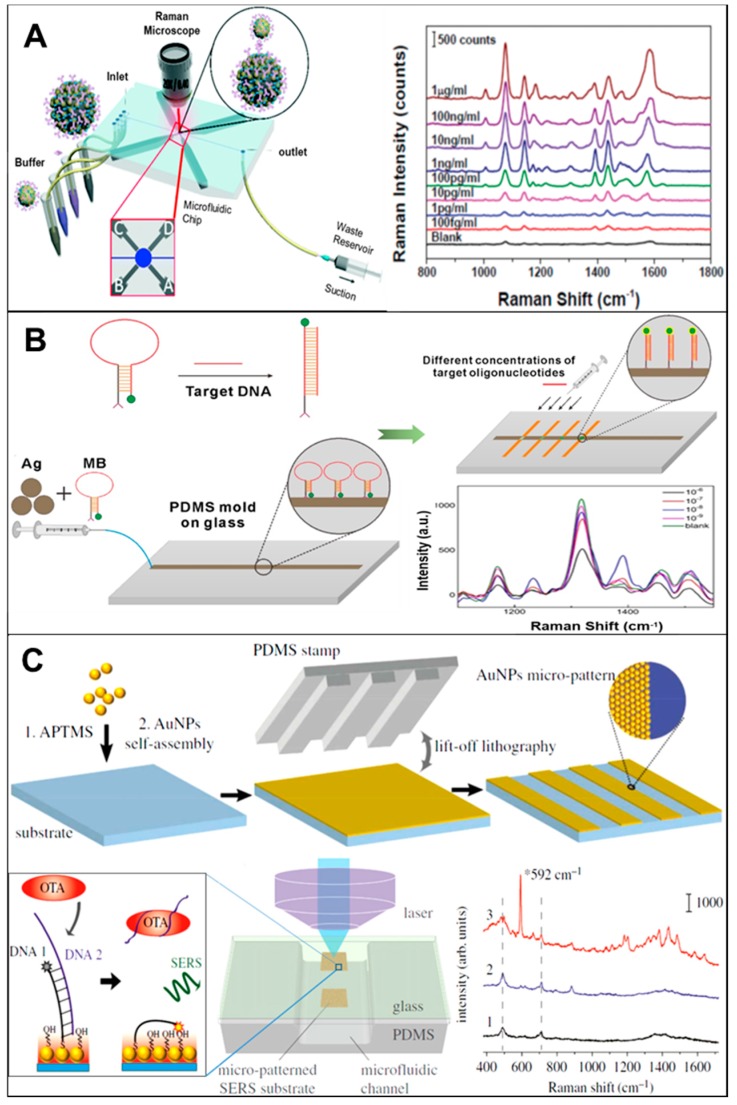
(**A**) Schematic illustration of the SERS-based immunoassay in a microfluidic chip for rabbit IgG detection (reprinted with permission from Royal Society of Chemistry [68]); (**B**) scheme depicting the working mechanism of the designed molecular beacon (top left) and the SERS spectra of the molecular beacon (MB) probes with different concentrations of target DNAs, (reprinted with permission from IOP Sciences [71]); (**C**) a schematic illustration of a SERS substrate with AuNPs is prepared using an APTMS-assisted surface-assembly method, then a polydimethylsiloxane (PDMS) stamp is brought in to make contact with the AuNPs layer, and the removal of the PDMS stamp lifts off the AuNPs and leaves the desired SERS-active pattern. SERS detection at the SERS-active substrate inside a microfluidic channel where SERS detection of ochratoxin A (OTA) using OTA aptamer took place (reprinted with permission from Royal Society of Chemistry [72]).

Multiple examples that use pre-synthesized NPs in microfluidic channels for label-free SERS detection can be found in the literature. These publications cover a broad range of NPs composition, sizes and shapes, as well as different applications such as food [59], healthcare [65], environment [31], or security [66]. We will present in this section some of the most recent reports, as well as a variated selection, in order to show the full potential of this approach. Mungroo et al., presented a combination of microfluidics-based chemometric analysis and Raman spectroscopy to detect the presence of bacteria in food material [67]. This procedure uses silver nanoparticles (AgNPs) and Triton X-100 to discriminate between eight different foodborne pathogens (*E. coli*, *S. typhimirium*, *S. enteritis*, *P. aeruginosa*, *L. monocytogenes*, *L. innocua*, *MRSA 35*, and *MRSA 86*). The microfluidic chip consisted of two inlets and one outlet, one mixing channel, and one reaction chamber, connected with a capillary pump. This model microfluidic chip, integrated with SERS and chemometric analysis, was able to effectively discriminate a series of peak assignments for each individual bacterial species in a label-free configuration. The use of principal component analysis (PCA) allows for the treatment of the high amount of acquired data in a more effective way. As a result, after pre-processing data for two of the species used in the study, detection limits down to seven colony forming units (CFUs) were achieved. In a further example, Yap et al., reported on the design and use of a microfluidic device with bifunctional (SERS and magnetic) particles for immunoglobulin G (IgG) detection, as shown in Figure 3A. It consists of a micromixer and an assay chamber able to perform multiple step assays for direct SERS detection on a microfluidic chip [68]. On-chip micro-mixing is performed with the help of four electromagnets controlled by a microcontroller unit. By switching a magnetic pin on and off, the magnetic nanoparticles are moved in the pattern of the flow on the microfluidic chip. An efficient mixing is capable of speeding up the assay time, as well as enhancing the signal for a magnetic nanoparticle-based immunoassay. The limit of detection (LOD) for SERS-based microfluidic immunoassay platform is down to 1 pg mL^−1^ for rabbit IgG protein. The micro-mixing on-chip improves the binding specificity by ~70% and detection time from 4 h to 80 min. This on-chip mixing and detection approach could be potentially utilized for point of care (PoC) diagnostics, as sample preparation, washing, and detection are all integrated in the same device. Zhang et al., demonstrated a magnetic-capture-based SERS assay for detection of microRNAs, in which miR-141 was used as target sequence and captured by hybridization reactions with complementary probes conjugated with Raman-active AuNPs and gold-coated paramagnetic nanoparticles (Au@MNPs) [69]. SERS response to the formation of hybridization complexes on Au surfaces revealed the RNA/DNA interactions involved in the specificity of miR-141 detection. The LOD was estimated in 100 fM, a 100-fold improvement over the LOD (10 pM) reported in a similar magnetic nanoparticle-based assay for DNA detection. The enhanced detection sensitivity is due to the plasmonic–magnetic core-shell structures that increase the probability of forming SERS hot spots. However, this system could be further improved by optimising the size and shape of nanoparticles to achieve a higher enhancement. The same researchers recently reported on an improved SERS substrate using the same type of NPs but coated with a silica layer, and using a modified detection scheme [70]. Potentially, this last generation of NPs that show improved LOD in real samples can later be combined with microfluidics. Abalde-Cela et al. demonstrated the possibility of using a simple microfluidic device for the multiplex detection of different analytes in real-time. [62] The use of a cross-shaped microfluidics device and of AgNPs as enhancing material showed a remarkable performance for the simultaneous ultra-detection of a mixture of four different analytes. By using this optofluidic SERS device, detection limits in flow down to the nM range were achieved.

### 3.2. Immobilized Nanoparticles (Continuous/Static Flow)

A different strategy for label-free detection using SERS in microfluidic channels is the use of pre-formed NPs, or the in-situ synthesis of NPs for their assembly in microfluidic channels. Several examples have been reported in the literature, such as the use of silver nanoprobes in microchannles [27], the detection of cancer biomarkers by a gold gradient assembly [73], or a CD-based SERS concentrator and amplifier [74]. Among the recent examples, we can highlight the work presented by Wang et al., a novel method for miRNA-21 detection through the combination of fluorescence and SERS spectroscopies, where 6-carboxyfluorescein (6-FAM) labelled on a molecular beacon (MB) is used [71]. The use of a microfluidic chip in this case allows for control of the injection flow, the mixing of target molecules and reaction time according to the substrate NPs and target concentration. Both fluorescence and SERS show the opposite changing trends for alteration in concentration of miRNA. As schematically illustrated in Figure 3B, the MB is immobilized on the SERS enhancing substrate formed by assembling Ag-NPs. The recognition of target miRNA is facilitated by specific binding site of MB. If target miRNA has a hairpin loop, then labelling of 6-FAM is only possible close to the Ag-NPs on the MB, leading to a weak fluorescence signal and a strong SERS signal. If the target miRNA has an unfolded hairpin structure then 6-FAM remains far from Ag-NPs, resulting in a strong fluorescence and weak SERS signal. The experimental results proved that the combination calibration curve is more precise for detection limits, resulting in improved sensitivity and dynamic range. In a different approach, Wang et al., also introduced the concept of a self-referencing mechanism that employed SERS molecular probes to attain one step detection of target bacteria for rapid and accurate diagnostics of water samples [75]. To improve the LOD for on-site detection system, a bacterial concentration mechanism based on nano-dielectrophoretic (Nano-DEP) enrichment was used during SERS signal acquisition on microfluidic platform. Only specific binding of nano-probes to targets will yield a detectable dual SERS signal (i.e., probe + target signals). The non-specific binding or no-binding will not yield dual signals in this process, because only the specially designed SERS probes, made from functionalized anisotropic nanoparticles, can generate enough enhancement to the SERS-detectable signal. This novel approach of multiplex self-referencing SERS pathogen detection offered high sensitivity (10^1^ CFU/mL) and strain level discrimination by measuring the superimposed SERS signatures with multiple characteristic peaks. The SERS spectra with superimposing of both detection can obtain in single step without washing step on the microfluidic system. The claim for the sensitivity and accuracy was checked against the gold slandered ELISA techniques. Furthermore, to improve the LOD up to 100 CFU/mL, this innovative microfluidic platform can be integrated with a separation and concentration system of Nano-DEP microfluidic. For the first time, Li et al., implemented nanoporous gold structures inside a microfluidic chip for increased plasmonic surface area [76]. These developed nanoporous gold disks (NPGDs) have 20-fold larger surface area than solid gold disk substrate as a result of its porosity. The SERS-active NPGD arrays exhibit a suitability for reproducible signal enhancement. Model dye (R6G) solutions ranging from 1 μM to 1 mM were used for quantitative evaluation of the performance of this SERS substrate in continuous-flow conditions (LOD = 5.16 nM). Remarkably, this sensor shows a quick (i.e., <2 s) label-free SERS detection on a microfluidic platform. The system was also tested against the neurotransmitter (DA; LOD = 32.4 nM)), and a physiological metabolite (urea; LOD = 0.67 mM). The obtained results show an improvement in LOD compared with other established LoC-SERS platforms for detection and quantification of biomolecules in urine screening and kidney function monitoring [77]. Furthermore, Wu et al. used the stamp transferring in soft lithography in PDMS with this approach, they successfully fabricated two-dimensional structures of gold nanoparticles with a line width of several micrometres [72]. They further bonded a PDMS cover with designed concave channels to the glass slide to make a microfluidic chip with an integrated SERS substrate. This chip is capable of on-site SERS detection by directly focusing the incident laser onto the inner surface of the glass slide directly through the rear of the slide (Figure 3C). This developed SERS substrate-embedded microfluidic chip can be fabricated with good reproducibility. The SERS performance of the fabricated micro-patterned substrate was evaluated by using Rhodamine 6G (R6G) as the SERS probe. This device is fabricated for in situ detection of desired SERS signal of Nile blue in the microchannel, with the limited distraction of impurities and background of bulk PDMS. The indirect SERS detection of OTA, a mycotoxin binding with aptamer, was successfully demonstrated. Also, Novara et al. presented a label-free two-step method for the quantification of miRNA using silver decorated porous silicon-PDMS membranes integrated into microfluidic chips [78]. Two bioassays based on a standard one-step and a new two-step hybridization approach were developed and compared. The surface modification approach was divided into two parts, where the first half was working as the recognition probe, and Raman label was introduced in the second half probe used in the second hybridization, which avoids any further mismatch of the target. The optimization was assisted by the SERS analysis at each functionalization step, which enabled the identification of the optimal probe density by variation of the salt and probe concentrations in the immobilization buffer, in order to maximize the hybridization events. The best results were achieved with the resonantly excited Cy3 dye, which was used for the analysis of different miR-222 concentrations. The LOD of detection for both the approaches of bioassay was 0.55 and 1.51 nM, respectively. The small RNA extracts of NSCLC H460 cells were analyzed in the microfluidic chips, which shows the feasibility of detection and quantitation of miRNAs in real biological samples using SERS. Finally, Gómez-Graña et al., reported the aqueous synthesis of uniform Au octahedral NPs using pre-synthesized single crystalline gold nanorods as seeds and butenoic acid as a mild reducing agent in aqueous medium [37]. This method also allows for a wide tunability of the Au octahedra size from 50 to 105 nm in side length. The assembly of these octahedra into 3D crystals in microfluidic devices and its SERS performance was demonstrated. These octahedral particles exhibit homogeneous SERS activity over large areas, which is a key parameter for quantitative analytical applications. The presented work also shows the potential and advantages of the seed-mediated uniform growth of SERS active materials in microfluidic channels and their controlled shape and size for different applications in SERS based biomedical detection devices.

### 3.3. Segmented Flow

**Figure 4:**
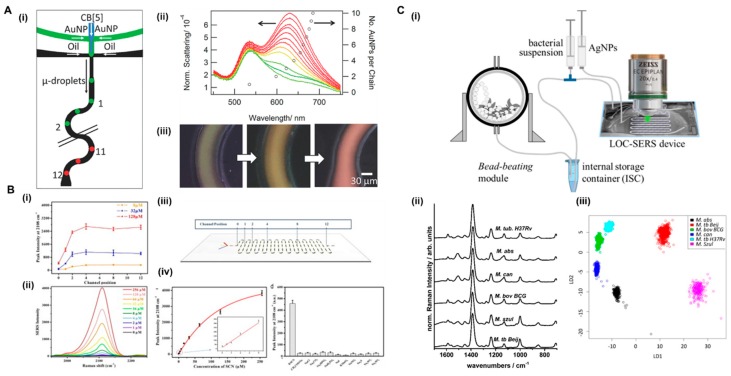
Microdroplets-based examples for label-free detection in microfluidics; (**A**) monitoring early-stage nanoparticle assembly in microdroplets by optical spectroscopy and SERS (i) schematic of microfluidic device used for microdroplet nanoparticle assembly experiments, (ii) dark-field scattering spectra at these positions are plotted in panel, and (iii) dark-field images from the start, middle, and end of the serpentine channel (reprinted with permission from Wiley [80]); (**B**) (i) plot of peak intensity for thiocyanate (SCN^−^) at 2108 cm^−1^ as a function of channel positions for the detection of 8, 32, and 128 μM SCN^−^, (ii) concentration-dependent SERS spectra for SCN^−^ detection; (iii) device positions for SERS measurements in microdroplets; (iv) plot of peak intensity at 2108 cm^−1^ as a function of SCN^−^ concentration (left) and comparison of peak intensity at 2108 cm^−1^ of Au@Ag NRs mixed with different anion ions (right) (reprinted with permission from Elsevier [53]) (**C**) LOC-SERS device for bacteria detection; (i) scheme of sample preparation, including the sample lysing module (bead-beating system) for the bacterial cell disruption, the internal storage container, the syringe pump system, and the droplet-based microfluidic device mounted to the microscope stage; (ii) mean spectra recorded from three batches of six different mycobacterium species; (iii) principal component analysis-linear discriminant analysis (PCA-LDA) model trained to separate the six species data plot using a hierarchical model (reprinted with permission from Mühlig [61]. Copyright 2016 American Chemical Society).

Several reports combining microdroplets-based strategies and Raman label-free detection have been published in the recent years. The use of microdroplets is beneficial as they act as individual microreactors, enhance fluid mixing and avoiding nanoparticle adsorption in the microfluidics channels, thus preventing the memory effect [79]. Several applications, analytes, and events have been demonstrated, such as the monitoring of nanoparticle assembly formation in microdroplets [80], presence of contaminants in water samples [81], tracing dyes and small atoms [82,83], or ultra-detection of drugs [30]. One of the most recent examples is that published by Popp and co-workers, in which they developed a LOC-SERS device able to differentiate six mycobacterial species (non-tuberculous versus tuberculous species) without any sample preparation [61]. A droplet-based device combined with chemometric analysis based on the signal of the cell wall of different bacteria was used for this purpose (Figure 4C). The fact that this device integrates a bead beating module for cell wall disruption avoids the sample preparation off-chip, and thus increases not only the efficiency and number of operations for detection, but also the safety of the analysis. The identification of different species of different mycobacterial groups using this LOC-SERS device showed an accuracy of 93.3%. In a different approach, Wu et al., also used a microdroplet-based approach for the detection of thiocyanate (SCN^−^) in real human body fluids (serum and saliva) [53]. First, the performance of the SERS enhancing nanomaterials was tested with standard SCN^−^ solutions, as SERS substrate Au@Ag core-shell nanorods (Au@Ag NRs) were used for enhancing the signal of the target molecule, as shown in Figure 4B. The Au@Ag NRs were encapsulated in microdroplets together with different concentrations of standard solutions of the analyte and the method revealed high specificity, reproducibility, and sensitivity. In order to support the latter affirmation, they compared the on-chip results with SCN^−^ detection on solid metal surfaces. The assay in droplets showed a LOD of 1 µM, with a linearity range up to 8 µM. This LOD is comparable with that detected off-chip in the solid substrates. Afterwards, they applied this method to the analysis of real samples from human body fluids, more specifically saliva and serum. The speed of analysis, as well as the reproducibility, were remarkably better by combining SERS and microdroplets. Further, the use of segmented flow prevented the memory effect in microfluidic devices because of the adsorption of nanoparticles in the channels walls when using continuous flow microfluidics. Demonstrating the applicability to different fields, Meier et al. used a microdroplets device for the monitoring of a chemical reaction [84]. They took advantage of microdroplets acting as microreactors for the study in real-time of organic reactions. Pre-synthesized Ag NPs were used as enhancing SERS material and encapsulated in microdroplets with reaction product precursors. Different detection points were chosen along the microfluidic channel after microdroplet formation to monitor the evolution of the reaction to form 2-aminothiazoles. Salmon et al. used SERS in combination with droplet microfluidics to track the formation of nanogold assemblies in microdroplets [80]. The microdroplet confined environment provided millisecond time resolution, allowing for the identification of a regime where dimer formation dominated (Figure 4A). In this example, the microdroplet environment also provides a faster mixing of the Au NPs and the cucurbit[5]uril (CB[5]), acting as molecular glue for the assembly formation. This approach shows potential for future applications such as nanoparticle isolation and sorting.

## 4. Bottlenecks and Future Outlook

Herein, we presented a broad range of examples and possible configurations for label-free detection using SERS in combination with microfluidics. Additional examples can be found in Table 1. As explained above, there are several considerations to take into account depending on the analytical problem to tackle. First, the design of the sensing material is of utmost importance, and the size, shape, composition, and architecture of the SERS substrate need to be planned accordingly. Further, the design, material, and operations need to be integrated in microfluidic devices for sensing have to be considered to be successfully combined with SERS substrates. Up to date, most of the reports used mainly Au, Ag, or Au/Ag NPs as SERS enhancing material, both as synthesized, as well as supported along the microfluidic channels. Microfluidic devices used in the papers explained along this review used PDMS or glass as fabrication materials. The most relevant advantages of this combination can be seen from two different points of view. For SERS, it is relevant that the detection can be done in an automated way, which allows for higher reproducibility than when compared with bulk SERS experiments. From the microfluidics point of view, the use of SERS allows ultra-detection, which is key when dealing with the small sample volumes handled in microfluidic channels. SERS, being a vibrational based technique, offers the possibility of label-free analysis, as demonstrated for several analytes and in different application fields (Table 1). However, the method is still limited by the difficulty of the analysis of complex Raman spectra, especially when looking for multiplex analysis. Usually, when doing multiplex analysis, the use of SERS tags is applied, which limits the use of the technique to non-label-free approaches. In order to use label-free SERS and take advantage of the high-throughput of microfluidics, it is necessary to implement chemometric tools for the deconvolutions and classification of the output spectra in an efficient manner. Further, the use of SERS as it is a relatively novel analytical technique, instrumentation development, miniaturization, and nanoparticle batch-to-batch reproducibility are still challenges that remain untackled when compared with most extended techniques used in combination with microfluidics, such as fluorescence. We believe that with the current booming of data science and its integration with lab-on-a-chip devices, the advantages that SERS spectroscopy offers over other analytical techniques can be further boosted for the label-free analysis of complex sensing challenges ahead. 

## Figures and Tables

**Table 1:** Additional examples of lab-on-a-chip (LOC)-surface-enhanced Raman scattering (SERS) based sensors for various targets. LOD—limit of detection.

SERS Substrate	Device Type	Strategy	Target	LOD	Refs.
Ag NPs	PDMS	Dispersed NPs continuous flow	Carboxyfluorescein-labelled DNA	0.5 µM	[85]
Fe_3_O_4_@Au NPs@Ag	PDMS	Dispersed NPs continuous flow	IgG	1 pg mL^−1^	[68]
Au@M NPs	PDMS	Dispersed NPs continuous flow	miRNA	100 fM	[69]
Ag NPs	PDMS	Dispersed NPs continuous flow	Foodborne bacteria (8 species)	7 CFU	[67]
Ag NPs	PDMS	Dispersed NPs continuous flow	Dyes	0.9 nM	[62]
Ag NPs	PDMS	Immobilized NPs continuous flow	miRNA	10^−8^ M	[71]
Nanoporous Au disks	PDMS	Immobilized NPs continuous flow	R6G DA Urea	5.16 nM 32.4 nM 0.67 mM	[76]
Ag Zn(OH)F@ZnS	Capillary	Immobilized NPs continuous flow	Bovine serum albumin (BSA)	1 pM	[86]
Au NPs	Glass	Immobilized NPs continuous flow	R6G	0.5 pM	[87]
Ag NPs	PDMS	Immobilized NPs static condition	*E. coli*	4.5 × 10^3^ cells/mL	[88]
Au NPs	PDMS	Immobilized NPs static condition	*E.coli* O157:H7	1 CFU/mL	[75]
Ag NPs	PDMS	Microdroplets	Mycobacteria species	93.8%	[61]
Ag NPs	PDMS	Microdroplets	2-aminothiazole		[84]
Au@Ag NRs	PDMS	Microdroplets	Thiocyanate	1 µM	[53]
